# Latent infection after spinal cord stimulation device implantation for complex regional pain syndrome: A case report

**DOI:** 10.1097/MD.0000000000033750

**Published:** 2023-05-12

**Authors:** Yu Min Ki, Hue Jung Park, Seung Hyun Yi, Woo Seog Sim, Jin Young Lee

**Affiliations:** a Department of Anesthesiology and Pain Medicine, Samsung Medical Center, Sungkyunkwan University, School of Medicine, Seoul, Korea; b Department of Anesthesiology and Pain Medicine, Seoul St. Mary’s Hospital, College of Medicine, The Catholic University of Korea, Seoul, Korea.

**Keywords:** complex regional pain syndrome, infection, latent, spinal cord stimulation

## Abstract

Spinal cord stimulation (SCS) is one of the invasive treatments of complex regional pain syndrome (CRPS). The positive effect has been observed for several years after implantation. However, infection is a common cause of SCS failure and device removal.

**Patient concerns:**

Here we describe a case of latent infection at the implantable generator pocket site 9 years after SCS implantation in a patient with CRPS.

**Diagnoses:**

A 52-year-old patient was diagnosed with type 1 CRPS. The right foot pain was intractable with standard treatments. SCS implantation was performed and SCS worked well without complication. Nine years later, the patient revisited due to pain, tenderness, and redness at the abdominal wall for 2 weeks. The right foot pain was maintained with NRS 4 for 8 years, and the stimulation stopped 1 year back. SCS infection was diagnosed.

**Interventions:**

The patient underwent SCS removal surgery.

**Outcomes:**

All SCS devices were removed successfully. The patient was discharged without any complications.

**Conclusions:**

While uncommon, infection after SCS implantation can occur even 9 years later. Immediate diagnosis, proper antibiotics, and surgical removal could be needed to prevent further spread of infection and better prognosis.

## 1. Introduction

Complex regional pain syndrome (CRPS) is characterized by spontaneously evoked pain, impaired motor function, and autonomic dysfunction with sensory and vasomotor disturbances. Owing to its complex pathophysiology, CRPS should be managed with multi-disciplinary treatment.^[1]^ To date, standard treatments (medications, intravenous regional block, sympathetic block, somatic nerve block, and central neuraxial block) along with physiotherapy have been used with varying degrees of efficacy, with neuromodulation using spinal cord stimulation (SCS) device being the next option.^[2]^ Electrical stimulation interferes with the transmission of neuropathic pain impulses from primary small-fiber afferents.^[3]^ SCS is made up of an implantable pulse generator (IPG) connected to one or more stimulating leads introduced into the epidural space. After a trial phase with successful stimulation, permanent leads are reintroduced to the same location in the epidural space and anchored, and pulse generator is implanted in the abdominal wall, gluteal area, or flank.^[4]^ The positive effect of SCS has been observed for several years after implantation in CRPS.^[1]^ However, possible complications include electrode dislocation, infection, dural puncture, and equipment failure.^[3]^ Infection is a common cause of SCS failure and device removal, which leads to further disability, and increased mortality and healthcare costs.^[4]^ Inoculation of IPG with microorganisms from the skin flora or contaminated aerosol is the most likely mechanism of SCS infection. Biofilm formation around bacteria protect them from the host’s defense and antibiotic penetration.^[4]^ Perioperative risk factors for infection include prolonged operative time and duration of trial phase over 5 days. Permanent implants following the trial phase have significantly lower rate of infection than in the trial phase because the leads of trial SCS system are externalized and microorganisms can enter the wound through this route.^[5]^ We report the case of infection at the IPG site in the right abdominal wall 9 years after SCS implantation.

## 2. Case presentation

**Figure 1.**
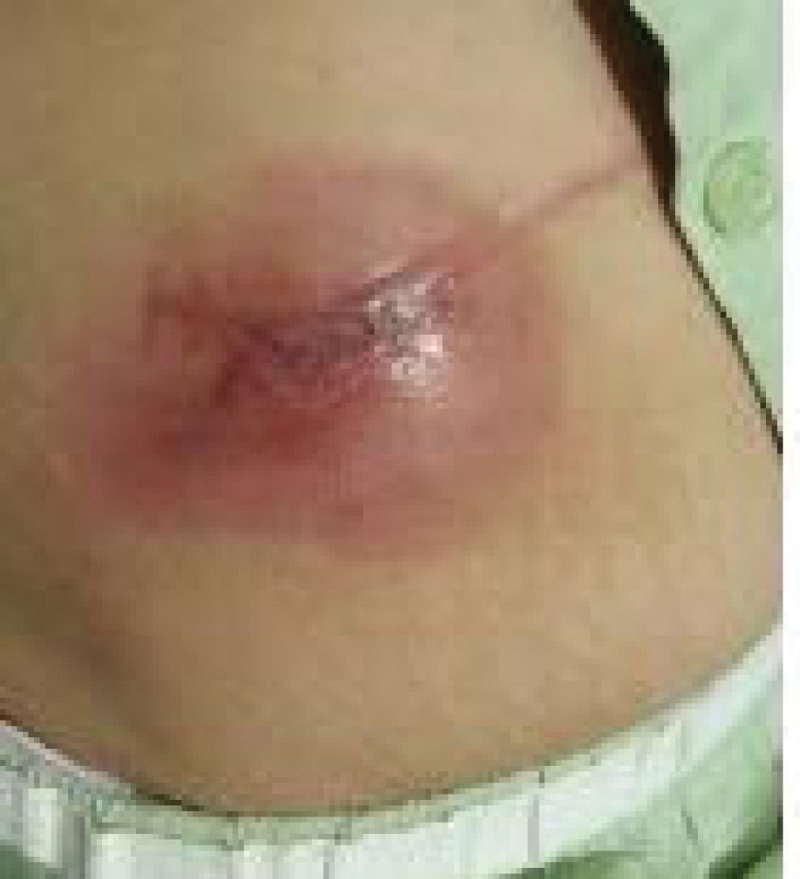
Infection in the abdominal wall after 1 month of SCS implantation. SCS = spinal cord stimulation.

**Figure 2.**
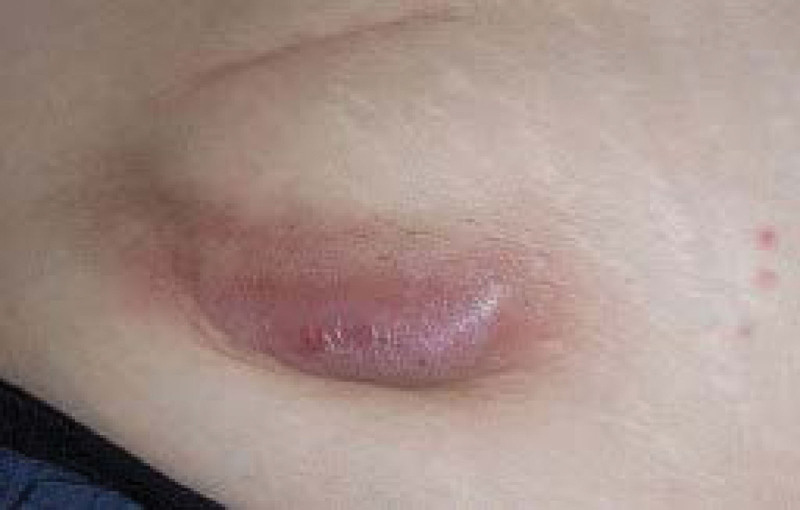
Infection in the abdominal wall after 9 years of SCS implantation. SCS = spinal cord stimulation.

This case report was approved by our departmental ethics committee (SMC 2022-10-094). A database from university pain clinic between December 2011 to May 2022 was retrospectively reviewed. A written consent was obtained from the patient. A 52-year-old female patient with body mass index of 25.6 kg/m^2^ was referred to our pain clinic with the right foot pain. She was diagnosed with plantar fasciitis at another hospital and underwent calcaneal spur reduction surgery 3 months back. Her foot pain persisted despite the surgery. She experienced stabbing, lancinating pain, and coldness: 9 on the numerical rate scale (NRS, 0 = no pain, 10 = worst pain imaginable). Allodynia, hyperalgesia, and atrophic changes were observed in the right foot, and the patient experienced functional impairment. Electromyography confirmed right lateral plantar neuropathy. She was diagnosed with type 1 CRPS. She had been treated with pregabalin, cetamadol, duloxetine, alprazolam, and oxycodone with limited effects. She underwent interventions including lumbar epidural block, caudal block, popliteal nerve block, and lumbar sympathetic ganglion block, with 10% to 20% pain relief for a few days. We decided to proceed with SCS trial. After sterile draping, through the paraspinal approach at the L3-4 level, the epidural space was confirmed using the loss of resistance technique under fluoroscopic guidance. The tip of lead was placed on the right side at the T12 level, and the procedure was finished after confirming the adequate stimulation of the pain site. During the trial period, pain score of the right foot significantly decreased to NRS 4-5, and the patient was satisfied with the effect; therefore, we decided to perform permanent implantation. After 4 days of the trial phase, permanent SCS implantation was performed. An IPG was inserted into the pocket in the right abdominal wall and connected to the lead through subcutaneous tunneling. SCS implantation was successful, and the patient was discharged without any other adverse effects. However, 1 month later, she presented redness, serous discharge, and wound dehiscence at the IPG site (Fig. 1). IPG wound infection was suspected, and wound culture showed *Staphylococcus aureus* and *Enterobacter aerogens*. She was administered cefazoline intravenously. We decided IPG removal for treating the infection. IPG was removed after disconnecting the extensor from the lead. Two weeks later, IPG was re-implanted at the new pocket in the right abdominal wall, which was further down from the previous site and the extensor was connected to the lead through the new tunnel of subcutaneous tissue. The pain was relieved to NRS 4, and subjective function of the right foot improved. After discharge, she visited our clinic as a routine follow-up for 5 months, and SCS worked well without complication. Nine years later, she revisited due to pain, tenderness, and redness at the IPG pocket for 2 weeks (Fig. 2). The right foot pain was maintained with NRS 4 for 8 years, and the stimulation stopped 1 year back. We decided to remove all SCS devices. During surgery, severe adhesions were observed inside the back muscles, tunneling site, and right abdominal wall. All SCS devices were removed successfully. She was discharged without any complications.

## 3. Discussion

The favorable efficacy of SCS for treating CRPS has been verified in several large cohort studies with evidence of high patient satisfaction, analgesic sparing effect, and pain relief.^[1,6]^ Continuous significant reduction in pain scores was reported during 8 consecutive years with SCS.^[1]^ SCS system generates a weak electrical field stimulating dorsal column of the spinal cord.^[7]^ It evokes peripheral paresthesia in the pain affected dermatome, leading to pain relief.^[1]^ The overall complication rate for SCS is 30% to 40%.^[3]^ Infection is one of the major complications with incidence of 2.4% to 10%.^[1,3]^ The most common site of infection is the IPG site, followed by leads, and lumbar incision sites.^[3,5]^ Median time to infection onset is 27 days (range 2–967) following implantation.^[3]^ Localized incisional pain and wound erythema were the common presenting signs in clinical exam.^[3]^ The incidence of epidural infection is 0.1%, but the diagnosis of epidural abscess may be delayed in the absence of neurological deficits.^[3]^ Early infections are mainly due to bacterial contamination during the surgical procedure. To the best of our knowledge, so far, there is no report of latent infection at 9 years of SCS implantation. The prior IPG infection may have been a risk factor for this latent infection; however, the secondary IPG was made at a new site, which was further down than the prior IPG location. The sources of implant-related infection can be classified as perioperative (bacterial colonization at the time of surgery), contiguous (through wound contamination) or hematogenous (spread of bacteria through the blood and lymph nodes).^[4]^ In our case, the patient had severe adhesions around the IPG site and SCS anchoring site in paraspinal space during the removal surgery. We suspect that there may have been chronic inflammation around the SCS device and it worsened by unknown causes, which lead to the clinical signs of infection at the IPG pocket.

Early recognition of SCS infections is crucial to prevent further complications such as meningitis, epidural abscess, and/or vertebral osteomyelitis. Superficial infections in the absence of systemic signs or symptoms should be treated with oral or intravenous antibiotics.^[3]^ Incision and drainage or system explant should be considered in significant systemic response of infection, which is characterized by erythema and induration extending > 5 cm from the wound edge, temperature > 38.5℃, heart rate > 110 beats/min, or WBC count > 12,000/µL.^[3]^ In our case, the patient did not have any systemic infection signs with stable vital signs. However, the stimulation was stopped, and she desired device removal; therefore, we proceeded with SCS explantation. The decision of SCS explantation or non-explantation salvage therapy must be arrived based on individual cases depending on the severity and location of infection, time of infection (acute or latent), patient characteristics (infection risk factors), and benefits of SCS. While uncommon, infection after SCS implantation can occur even 9 years later. Immediate diagnosis, proper antibiotics, and surgical removal could be needed to prevent further spread of infection and better prognosis.

## Author contributions

**Data curation:** Jin Young Lee.

**Investigation:** Yu Min Ki, Seung Hyun Yi, Jin Young Lee.

**Supervision:** Hue Jung Park, Woo Seog Sim.

**Validation:** Jin Young Lee.

**Writing – original draft:** Yu Min Ki, Hue Jung Park, Jin Young Lee.

**Writing – review & editing:** Yu Min Ki, Hue Jung Park, Jin Young Lee.
